# An 8-gene diabetes-related signature predicts survival and immunotherapy response in breast cancer

**DOI:** 10.1016/j.clinsp.2026.100986

**Published:** 2026-05-09

**Authors:** Xin Jiang, Jianyun Yin, Yingying Tong, Jiayan Li, Xiang Ren, Changtai Zhu

**Affiliations:** a Department of Transfusion Medicine, Shanghai Sixth People's Hospital Affiliated to Shanghai Jiao Tong University School of Medicine, Shanghai, China; b College of Fisheries and Life Science, Shanghai Ocean University, Shanghai, China; c Department of Thyroid Breast Surgery, Kunshan Affiliated Hospital of Nanjing University of Chinese Medicine, Kunshan, China

**Keywords:** Breast cancer, Diabetes, Prognostic signature, Single-cell sequencing

## Abstract

**Objective:**

To develop a signature of Diabetes-Related Genes (DRGs) using data from the Cancer Genome Atlas (TCGA) and Gene Expression Omnibus (GEO) databases to predict the prognosis of Breast Cancer (BRCA) patients and identify potential therapeutic targets.

**Methods:**

A list of DRGs was sourced from the GeneCard database. Analyses of differential expression and consensus clustering were conducted to identify DRGs, and Cox regression and Least Absolute Shrinkage and Selection Operator (Lasso) regression were used to construct prognostic risk signatures based on DRGs. High- and low-risk groups were classified using median risk scores, and nomogram plots were created to visualize prognostic signatures. The relationship between immune infiltration, chemotherapeutic agents, and risk scores was evaluated, along with the expression of DRGs in individual immune cells.

**Results:**

A total of 1231 RNA-seq samples (cancer: 1118, paraneoplastic: 113), 1046 clinical data, and 965 DRGs were obtained from the databases. Differential expression and consensus clustering analyses identified 224 differentially expressed DRGs. Cox regression and Lasso regression analyses led to the establishment of a prognostic signature based on 8 DRGs. Kaplan-Meier curves demonstrated that patients in the low-risk group had a significantly better prognosis compared to those in the high-risk group (p < 0.001). The calibrated curves of the nomogram at 1-, 2-, 3-, and 5-years all aligned with the diagonal, indicating that our nomogram has high predictive performance. Immune infiltration analysis showed significant correlations between our constructed signature and the relative abundance of immune cells (p < 0.05). Single-cell data analysis revealed that DRGs were predominantly expressed in CD4+ conventional T-cells and regulatory T-cells.

**Conclusion:**

This study has successfully developed a prognostic signature for DRGs, which can be utilized as an efficient tool for risk stratification and prognostic prediction in BRCA patients. Additionally, this research offers a novel basis for investigating potential immunotherapy targets for BRCA patients.

## Highlights

• First diabetes-related 8-gene signature for breast cancer prognosis and immunotherapy guidance.
• Outperforms clinical variables (AUC=0.837) in predicting survival and stratifying risk.
• Linked to immune microenvironment – high expression in CD4+ T-cells and regulatory T-cells.
• Predicts chemotherapy sensitivity, identifying potential drugs for high- vs. low-risk patients.
• Provides a translational bridge between diabetes biology and precision oncology in breast cancer.

## Introduction

Breast Cancer (BRCA) is a significant health concern for women. Its incidence has been steadily increasing, surpassing lung cancer as the most common and deadliest cancer among females.^1^ Treatment options for BRCA include conventional chemotherapy, radiotherapy, surgery, and emerging therapies such as cell cycle management, signaling pathways, molecularly targeted therapies, monoclonal antibodies, antibody-drug conjugates, and immunotherapy.^2^, ^3^, ^4^, ^5^ While early diagnosis and multiple therapeutic approaches have improved prognosis and survival rates for BRCA patients,^6^^,^^7^ the risk of distant metastasis to organs like bone, brain, lungs, and liver remains a major cause of mortality.^8^ The risk factors for BRCA are diverse and complex, including gender,^9^ obesity,^10^ diabetes,^11^, ^12^, ^13^ genetic mutations, aging, familial inheritance, estrogen levels, and unhealthy lifestyles.^8^ Previously considered an age-related disease, diabetes is now being diagnosed in younger individuals, including children.^14^^,^^15^ Diabetes mellitus is a metabolic disorder characterized by hyperglycemia, resulting from impaired insulin action or secretion defects.^16^ Its development involves complex interactions between immune system disorders, familial genetics, and environmental factors.^17^, ^18^, ^19^ Furthermore, studies showed that diabetes may affect various organs, including the female breast, in different ways, such as metabolism, inflammation, and hormonal effects.^20^^,^^21^

The relationship between diabetes and Breast Cancer (BRCA) has been established.^22^^,^^23^ On one hand, up to 15% of BRCA patients have type II diabetes.^24^ Some studies have shown a high correlation between type II diabetes and a high risk of breast cancer,^20^^,^^21^ while women with type I diabetes have a 10 percent lower risk of breast cancer.^25^ On the other hand, diabetic BRCA patients have shown significantly worse overall and disease-specific survival compared to non-diabetic BRCA patients.^26^, ^27^, ^28^, ^29^ Additionally, there are notable differences in the treatment options chosen for BRCA patients with and without diabetes.^30^ However, the exact mechanism behind the association between diabetes and BRCA remains unclear, and there is a lack of standardized treatment protocols for patients with both conditions. The risk association between diabetes and BRCA has garnered increasing attention, highlighting the urgent need for comprehensive and in-depth studies on their relationship. Despite research suggesting that Diabetes-Related Genes (DRGs) may influence breast cancer progression,^31^^,^^32^ systematic biomarker signatures constructed based on DRGs that can be used to guide prognostic assessment and individualized treatment of BRCA patients are still lacking. Our study aims to fill this critical knowledge gap. In this study, the authors aim to develop a new signature of DRGs using data from the TCGA and GEO databases to predict the prognosis of BRCA patients. Furthermore, the authors will investigate the expression of immune infiltration and prognostic signatures at the individual immune cell level, providing a potential theoretical basis for immunotherapy in BRCA patients.

## Materials and methods

### Data collection and processing

RNA-seq data and clinical data for Breast Cancer (BRCA) were obtained from the Cancer Genome Atlas (TCGA) database (https://portal.gdc.cancer.gov/repository) and the Gene Expression Omnibus (GEO) database (https://www.ncbi.nlm.nih.gov/geo/). Perl scripts (version 5.32.1.1) were used to separate and collate the data. Gene-level copy number data for TCGA-BRCA were downloaded from the UCSC Xena database (https://xena.ucsc.edu/) and processed using Perl scripts. Clinical data that were missing and had a follow-up time of <30-days were deleted. A list of 964 Diabetes-Related Genes (DRGs) was searched and downloaded from the GeneCard database (https://www.genecards.org/) with a correlation coefficient higher than 0.4 as a screening criterion. The GEO dataset GSE10893-GPL887 (https://www.ncbi.nlm.nih.gov/geo/query/acc.cgi?acc=GSE10893) was integrated and analyzed with the TCGA-BRCA data. The TCGA-BRCA data served as the training set for feature building, while GSE10893-GPL887, GSE159956 (https://www.ncbi.nlm.nih.gov/geo/query/acc.cgi?acc=GSE159956), and GSE18229-GPL887 (https://www.ncbi.nlm.nih.gov/geo/query/acc.cgi?acc=GSE18229) served as the validation set for feature building. This study follows the Transparent Reporting of a multivariable prediction model for Individual Prognosis or Diagnosis (TRIPOD) reporting guideline.

### Analysis of differential expression and copy number

Transcripts per kilobase of exon model per million mapped reads (TPM) type data were extracted from the collated RNA-seq data using Perl scripts. The first step was to take intersections with 964 diabetes-related genes, followed by diabetes-related differential gene analysis of the TPM data, which was performed using the “limma” package (Ritchie et al., 2015) of the R software (version 4.3.1). The analysis included a filter of |log2 fold change (FC)| >1, False Discovery Rate (FDR) < 0.05. The results of the differential analysis were visualized and presented as volcano plots using the “ggplot2” package.

The transcriptome data of TCGA-BRCA and GSE10893-GPL887 were integrated using the “limma” and “sva” packages, and the ComBat function from the “sva” package was used to remove platform differences and batch effects. The expression matrices of the differential genes were extracted from the integrated transcriptome data based on the results of the differential analysis. The expression matrices were then subjected to one-way Cox regression analysis using the “survival” and “survminer” packages. Forest plots were drawn to visualize the results of the one-way Cox regression analysis.

Based on the results of the one-way Cox regression analysis, the network relationship information and node attributes were calculated using the “psych” and “reshape2” packages. The network relationship map of DRGs was drawn using the “igraph” and “RColorBrewer” packages.

The collated gene-level copy number data were plotted as a histogram of gene copy number frequency changes using the “barplot()” function of the “graphics” package in R. The gene-level copy number data were then integrated with the gene annotation information using a Perl script. The integrated data were plotted as a circle plot of copy number frequency changes using the “RCircos” package.

### Consensus clustering analysis of DRGs

Firstly, the expression matrix of the genes identified through one-way Cox regression analysis was extracted from the overall gene expression matrix. This specific matrix was then processed, and the genes were classified using the “limma” and “ConsensusClusterPlus” packages. The maximum number of subtypes was set to 9, and the results of each subtype were plotted separately. The optimal outcome was selected based on the subtype map, and a clinical correlation heatmap was generated using the “pheatmap” package in conjunction with TCGA-BRCA clinical information.

To assess the significance of the subtypes, three different data dimensionality reduction analyses were performed. These included Principal Component Analysis (PCA) using the prcomp() function from the “Stats” package, t-distributed Stochastic Neighbor Embedding (t-SNE) using the “Rtsne” package, and Uniform Manifold Approximation and Projection (UMAP) using the “umap” package. The results of these analyses were visualized using the “ggplot2” package.

Survival analysis was conducted to determine if there were differences in survival between the subtypes. This analysis utilized the “survival” and “survminer” packages. Additionally, differential expression analysis was performed to examine whether the genes identified through Cox regression analysis showed differential expression among the subtypes. The “limma” and “reshape2” packages were used for this analysis, and the results were presented as box plots using the “ggpubr” package.

Furthermore, Gene Set Variation Analysis (GSVA) was conducted using the “limma” and “GSVA” packages to identify differential pathways between the gene sets of the optimal subtypes. The results of the pathway differential analysis were visualized as a heat map using the “pheatmap” package. To assess the variance in the relative abundance of different immune cell types among the fractions, the authors conducted single-sample Gene Set Enrichment Analysis (ssGSEA) using the “limma”, “GSEABase”, and “GSVA” packages. Additionally, the authors utilized the “ggpubr” package to generate immune cell infiltration box plots for each fraction.

### Construction of A prognostic signature for DRGs

To construct a prognostic signature for DRGs, the authors initially performed Lasso regression analysis based on one-way Cox regression using the “glmnet” package. This step helped us identify and eliminate genes that may lead to overfitting. Subsequently, multifactorial Cox regression analyses were conducted using the “survival” and “survminer” packages to further refine and determine the prognostic characteristics of diabetes-associated BRCA.

To calculate the risk score for each BRCA patient, the authors used the following formula$:\;\text{Risk}\;\text{score}=\sum_{i=1}^{n}\left(\text{coefficient}\;\text{RNAn}\;\times \;\text{RNAn}\;\text{expression}\right)$; where “I” represents the serial number of all genes in the prognostic signature, and “n” represents the total number of genes in the prognostic signature.

After calculating the risk score for each patient, the authors categorized TCGA-BRCA patients into high-risk and low-risk groups based on the median risk score. To validate the prognostic value of the constructed risk profiles, the authors utilized the “survival” and “survminer” packages to perform Kaplan-Meier survival analysis for patients in the two risk groups. Additionally, the authors employed the “timeROC” package to conduct Receiver Operating Characteristic (ROC) curve analysis and the “pheatmap” package to generate risk scatterplots for the patients.

### Validation of the prognostic signature of DRGs and creation of a nomogram

To validate the applicability and stability of the prognostic signature constructed using DRGs, the authors independently validated the signature using the GEO datasets GSE10893-GPL887, GSE159956, and GSE18229-GPL887. Similar to validating the prognostic value of the risk profile, the authors calculated the risk score for all patients using the same formula, classified patients into high and low-risk groups based on the median risk score, and performed Kaplan-Meier survival analyses using the “survival” and “survminer” packages for patients in the two risk groups. The authors also conducted ROC curve analyses using the “timeROC” package and plotted risk scatterplots for the patients using the “pheatmap” package.

To determine whether our constructed DRGs' prognostic traits can serve as independent risk factors for predicting the prognosis of BRCA patients, the authors performed single and multifactorial Cox regression analyses using clinical traits, including risk scores, with the “survival” package. Additionally, to fully explore the prognostic value of our constructed DRGs prognostic traits, the authors created nomogram plots based on the “rms” package and plotted 1-, 2-, 3-, and 5-year calibration curves to assess the accuracy of the nomogram plots in predicting prognosis.

### Immune infiltration analysis

In the tumor microenvironment, our focus lies on the role of immune cells. To accurately assess the composition of immune cells in the tumor microenvironment, the authors utilized the R software to load the official source code provided by CIBERSORT. This involved specifying the benchmark database file (LM22.txt), which contains marker genes for 22 immune cells, downloaded from the CIBERSORT official website (https://cibersortx.stanford.edu/). The authors performed CIBERSORT immune cell infiltration analysis. Subsequently, the authors created violin plots to visualize the differences in relative abundance of immune cells between the high and low risk groups using the “vioplot” package. Additionally, the authors generated correlation heatmaps of immune cells using the “corrplot” package and correlation heatmaps between DRGs and immune cells using the “ggplot2” package.

### Drug sensitivity analyses

With advancements in biotechnology, tumor biotherapies such as tumor molecular targeted therapy, tumor immunotherapy, tumor gene therapy, and tumor stem cell-related therapy have become more mature. However, these therapies still have significant limitations, and chemotherapy remains the most effective tumor therapy throughout the course of cancer treatment. Therefore, the authors utilized the “oncoPredict” package to predict the sensitivity of chemotherapeutic agents in both the high and low-risk groups. The predictive analysis of the “oncoPredict” package is based on drug response information obtained from the Genomics of Drug Sensitivity in Cancer (GDSC) database.

### Single-cell data analysis in tumor immune single-cell hub-2 (TISCH2)

TISCH2 (http://tisch.comp-genomics.org/home/) is a single-cell sequencing database that focuses on the tumor microenvironment. To study the expression of our constructed prognostic traits at the individual cell level, the authors performed single-cell data analysis using the TISCH2 database.

Firstly, the authors selected GSE110686 for single-cell data analysis. The authors used UMAP as a data dimensionality reduction analysis method to cluster the cells. The authors then annotated and counted the number of different cells based on cell surface-specific markers. Secondly, the authors integrated the expression of featured genes from the database with the clustering results and visualized them in the form of a cluster diagram.

### Statistical analysis

The authors performed statistical analyses using R software (version 4.3.1) and Perl scripts (version 5.32.1.1). Survival analyses were conducted using the Kaplan-Meier method and the log-rank test. Prognostic characteristics were constructed using single- and multifactor Cox regression analyses, as well as Lasso regression analyses. Results were considered statistically significant when the two-sided p-value was less than 0.05.

## Results

### Analysis of differential expression and copy number

**Fig. 1:**
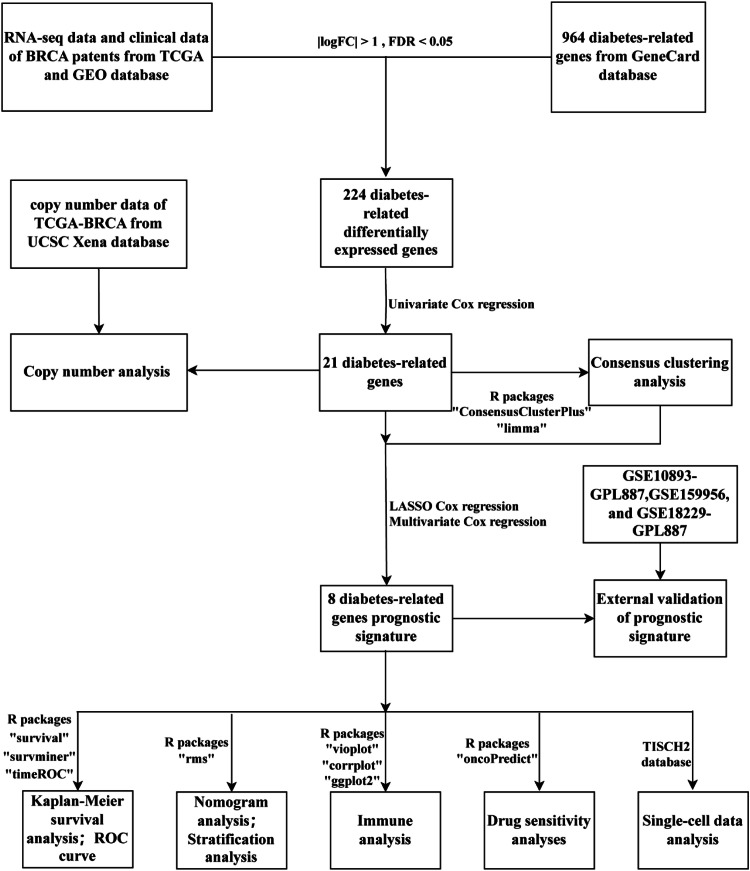
Overall flowchart of the study. BRCA, Breast cancer; TCGA, The cancer genome atlas; GEO, Gene expression omnibus; ROC, Receiver operating characteristic; TISCH2, Tumor immune single-cell hub-2.

**Fig. 2:**
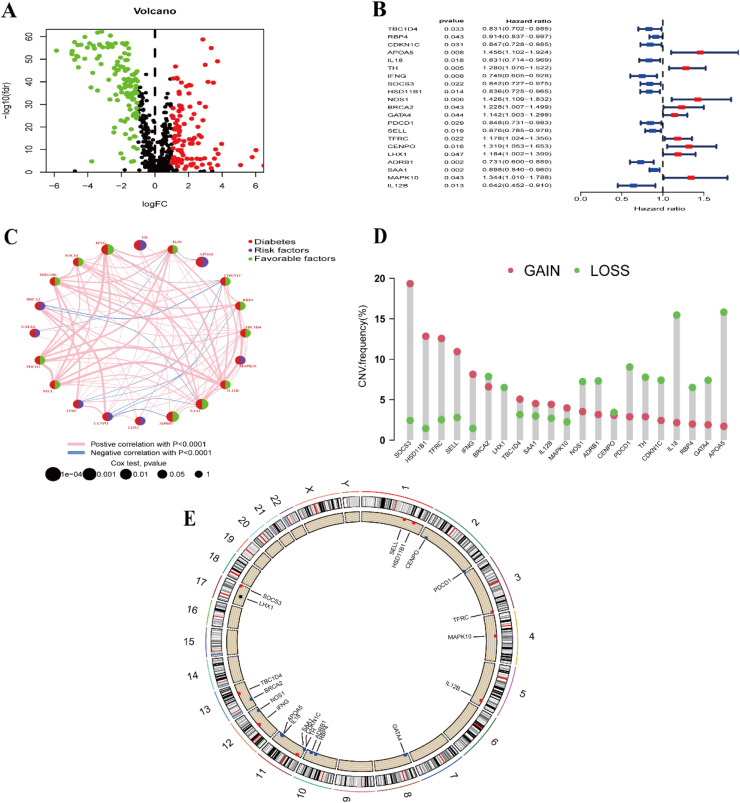
Differential and copy number analyses of diabetes-related genes. (A) Volcano plot of diabetes-related differential genes with screening thresholds set at FDR <0.05 and |log2FC| >1. (B) Forest plot of one-way Cox regression. (C) Network correlation plot of diabetes-related genes. Pink represents positive correlations and blue represents negative correlations. (D) Histogram of gene copy number frequency changes. Red color represents increasing frequency, and green color represents decreasing frequency. (E) Circle plot of gene copy number frequency changes. Red dots represent an increase in gene copy number frequency greater than the decrease; blue dots represent a decrease in gene copy number frequency greater than the increase, and the outer circle indicates the location of the gene on the chromosome. FDR, False Discovery Rate; FC, Fold Change.

Fig. 1 is a flowchart of our entire work, and Supplementary Table 1 presents the clinical information of the data the authors utilized. The authors extracted a total of 592 expression data for DRGs from RNA-seq data in TPM format. Differential analysis identified 224 diabetes related Differentially Expressed Genes (DEGs), as depicted in the volcano plot (Fig. 2A). Among these, 101 genes were up-regulated, while 123 genes were down-regulated. Using forest plot visualization (Fig. 2B), one-way Cox regression analysis identified 21 DRGs from the 224 diabetes related DEGs.

Examining the DRG network relationship map (Fig. 2C), it is evident that TBC1D4, RBP4, CDKN1C, IL18, IFNG, SOCS3, HSD11B1, PDCD1, SELL, ADRB1, SAA1, and IL12B act as favorable and protective factors for BRCA. Conversely, APOA5, TH, BRCA2, GATA4, TFRC, CENPO, LHX1, and MAPK10 are unfavorable risk factors. Additionally, the graphs allow us to observe whether the genes are positively or negatively correlated with each other. Notably, NOS1 does not exhibit any associations with the other genes.

The histogram of gene copy number frequency changes (Fig. 2D) and the circular plot of gene copy number frequency changes (Fig. 2E) clearly illustrate that the frequency of copy number increases for genes such as SOCS3, HSD11B1, and TFRC have a higher frequency of copy number increases than decreases, while genes such as BRCA2, NOS1, and ADRB1 (except LHX1) have a higher frequency of copy number decreases.

### Consensus clustering analysis of DRGs

**Fig. 3:**
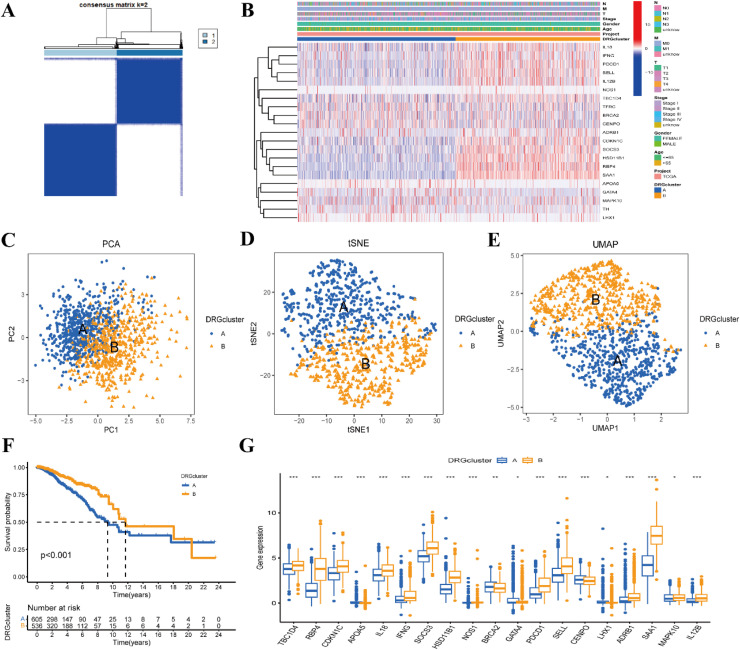
Consensus clustering analysis of diabetes-related genes. (A) Consensus clustering plot for clustering number k = 2. (B) Clinical correlation heatmap of phenotypes. (C‒E) 2D scatter plots of three data downscaling analyses of principal component analysis (PCA), t-distributed stochastic neighbor embedding (t-SNE), and uniform manifold approximation and projection (UMAP), respectively. (F) Kaplan-Meier survival curves of patients with clustering A and B. (G) Boxplots of differential expression of diabetes-related genes. * p < 0.05; ** p < 0.01; *** p < 0.001. DRG, diabetes-related genes.

Among the consensus clustering results with a clustering number k of 2‒9, the best results were obtained when k = 2. Fig. 3A illustrates the clustering graph for k = 2. The authors designated the two subtypes with k = 2 as subtypes A and B. The clinical correlation heatmap of subtypes (Fig. 3B) demonstrates significant differences in the expression levels of DRGs between subtypes A and B. The 2D scatter images of PCA (Fig. 3C), t-SNE (Fig. 3D), and UMAP (Fig. 3E), which represent three different data downscaling analyses, all indicate a clear distinction between subtypes A and B. Additionally, Kaplan-Meier survival analysis was conducted on patients with subtypes A and B. As shown in Fig. 3F, the survival rate of patients with subtype B was significantly higher than that of patients with subtype A (p < 0.001). The combined positive results from the clustering map, clustering heat map, data downscaling analysis, and survival analysis provide strong validation for the accuracy and stability of our clustering. Furthermore, the boxplot of gene differential expression (Fig. 3G) reveals significant differences in the expression profiles of the DRGs TBC1D4, RBP4, CDKN1C, APOA5, IL18, IFNG, SOCS3, HSD11B1, NOS1, BRCA2, GATA4, PDCD1, SELL, CENPO, LHX1, ADRB1, SAA1, MAPK10, and IL12B between subtypes A and B, as identified by one-way Cox regression analysis. This multi-level verification strategy effectively reduces the risk of bias in signature construction and provides methodological assurance for the robustness of subsequent prognostic signatures.

**Fig. 4:**
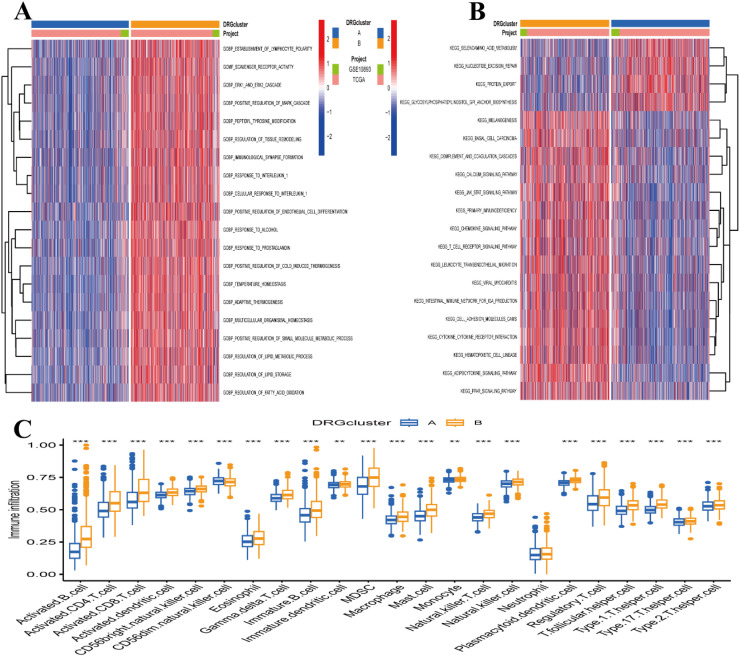
Gene Set Variation Analysis (GSVA) and single sample gene set enrichment analysis (ssGSEA) analyses of clustering diabetes-related genes. (A) Heatmap of GSVA analyses using the gene ontology (GO) gene set. (B) Heatmap of GSVA analyses using the kyoto encyclopedia of genes and genomes (KEGG) gene set. Blue represents clustering A, orange represents clustering B, green represents the GSE10893 dataset, and pink represents the TCGA-BRCA dataset. (C) ssGSEA analysis of the immune infiltration box plot of different immune cells between clustering A and B. * p < 0.05; ** p < 0.01; *** p < 0.001. GOBP, gene ontology biological process; DRG, diabetes-related genes.

And then, the authors performed GSVA analysis for Clustering A and Clustering B. The heatmap revealed that the differential signaling pathways analyzed between A and B were primarily related to the Gene Ontology (GO) gene set. In Fig. 4A, the authors display the top 20 pathways.

For the Kyoto Encyclopedia of Genes and Genomes (KEGG) gene set (Fig. 4B), the differential signaling pathways analyzed between A and B were mainly related to Glycosylphosphatidylinositol (GPI) anchor biosynthesis, JAK-STAT signaling pathway, primary immunodeficiency, T-cell receptor signaling pathway, and PPAR signaling pathway, etc.

Additionally, the authors utilized ssGSEA analysis to calculate the relative abundance of different immune cell types between subtypes A and B (Fig. 4C), except for neutrophils.

### Construction of a prognostic signature for DRGs

**Fig. 5:**
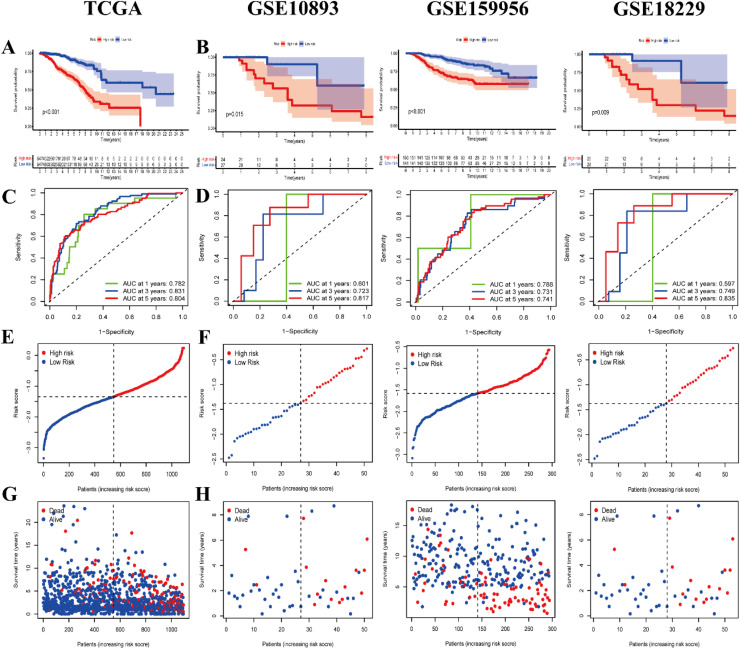
Kaplan-Meier survival analysis and receiver operating characteristic (ROC) curve analysis of the training and validation sets. (A) Kaplan-Meier survival curves of the training set TCGA-BRCA. (B) Kaplan-Meier survival curves of the validation sets GSE10893, GSE159956 and GSE18229. (C) ROC curves for the training set TCGA-BRCA. (D) ROC curves for validation sets GSE10893, GSE159956 and GSE18229. (E, G) Risk scatter plots of training set TCGA-BRCA. (F, H) Risk scatter plots of validation sets GSE10893, GSE159956 and GSE18229.

Lasso regression analysis eliminated 7 overfitting genes in addition to the ones identified through one-way Cox regression analysis (Supplementary Fig. 1A‒B). A total of 14 DRGs were subsequently identified. Further multifactorial Cox regression analysis was conducted to screen and identify the prognostic signatures of the DRGs, as presented in Supplementary Table 2. The prognostic signatures consisted of eight DRGs: TBC1D4, RBP4, CDKN1C, TH, IFNG, NOS1, TFRC, and ADRB1. Survival analysis demonstrated (Fig. 5A) that the low-risk group had a significantly better prognosis compared to the high-risk group (p < 0.001). The Area Under the ROC Curve (AUC) at 1-, 3-, and 5-years was 0.782, 0.831, and 0.804, respectively (Fig. 5C). Additionally, the risk scatter plots of TCGA-BRCA patients (Fig. 5E, G) exhibited a positive correlation between the risk scores and patient mortality rates. As the risk score increased, more patients tended to experience mortality.

### Validation and nomogram development for prognostic signatures of DRGs

**Fig. 6:**
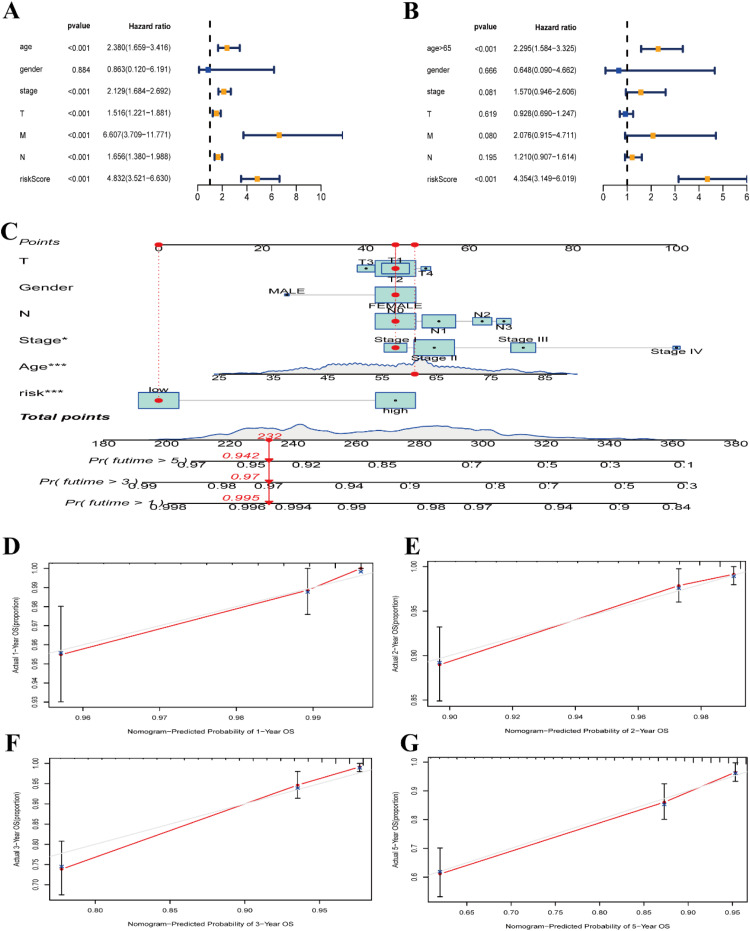
Creation and validation of column line plots. (A) Forest plot of one-way Cox regression analysis for each clinical trait. (B) Forest plot of multifactor Cox regression analysis for each clinical trait. (C) Column line plots created based on diabetes-related prognostic traits. (D) Column line plots calibration curves at 1-, 2-, 3-, and 5-years. *p < 0.05; ***p < 0.001.

The prognostic signatures of DRGs were validated, and a nomogram diagram was established. The independent validation cohorts, GEO datasets GSE10893-GPL887, GSE159956, and GSE18229-GPL887, were subjected to Kaplan-Meier survival analysis, confirming their validity (Fig. 5B). The low-risk groups in all three independent validation sets, GSE10893 (p = 0.015), GSE159956 (p < 0.001), and GSE18229 (p = 0.009), exhibited significantly better prognosis compared to the high-risk group. ROC curves and risk scatter plots were generated for each independent validation set, similar to the training set TCGA-BRCA. The AUCs at 1-, 3-, and 5-years for GSE10893 were 0.601, 0.723, and 0.817, respectively. For GSE159956, the AUCs at 1-, 3-, and 5-years were 0.788, 0.731, and 0.741, respectively. Lastly, for GSE18229, the AUCs at 1-, 3-, and 5-year were 0.597, 0.749, and 0.835 (Fig. 5D). The lower AUC at 1-year may essentially be due to short-term prognosis being influenced by random factors that cannot be captured by the signature, while molecular markers are better at predicting the intrinsic progression trajectory of the disease. This phenomenon is common in tumor/chronic disease prognosis studies. The risk scatter plots (Fig. 5F, H) of all three independent validation sets, GSE10893, GSE159956, and GSE18229, exhibited the same trend as the training set TCGA-BRCA, with the risk scores positively correlated with the mortality rates of BRCA patients. Furthermore, the patients' mortality rates increased gradually with higher risk scores. Both one-way Cox regression analyses (Fig. 6A) and multifactorial Cox regression analyses (Fig. 6B) associated with clinical traits demonstrated the significance of age (p < 0.001) and risk score (p < 0.001). This indicated that our traits were independent and could serve as stand-alone prognostic risk factors, regardless of other clinical traits. The results of the multivariate ROC curve analysis of risk scores and other clinical traits indicate that our prognostic model (AUC = 0.837) is significantly superior to age (AUC = 0.623), gender (AUC = 0.497), stage (AUC = 0.664), T-stage (AUC = 0.604), M-stage (AUC = 0.533), and N-stage (AUC = 0.649) (Supplementary Fig. 2A). Additionally, a nomogram plot based on DRGs prognostic traits (Fig. 6C) further confirmed the meaningfulness of risk score as a prognostic risk factor (p < 0.001).

The 1-, 2-, 3-, and 5-year calibration curves of the column line plots (Fig. 6D‒G) all nearly coincided with the diagonal line, suggesting that our column line plots have a high degree of accuracy in terms of their prognostic predictive power for BRCA patients.

### Validation of prognostic signatures and immune infiltration analysis

**Fig. 7:**
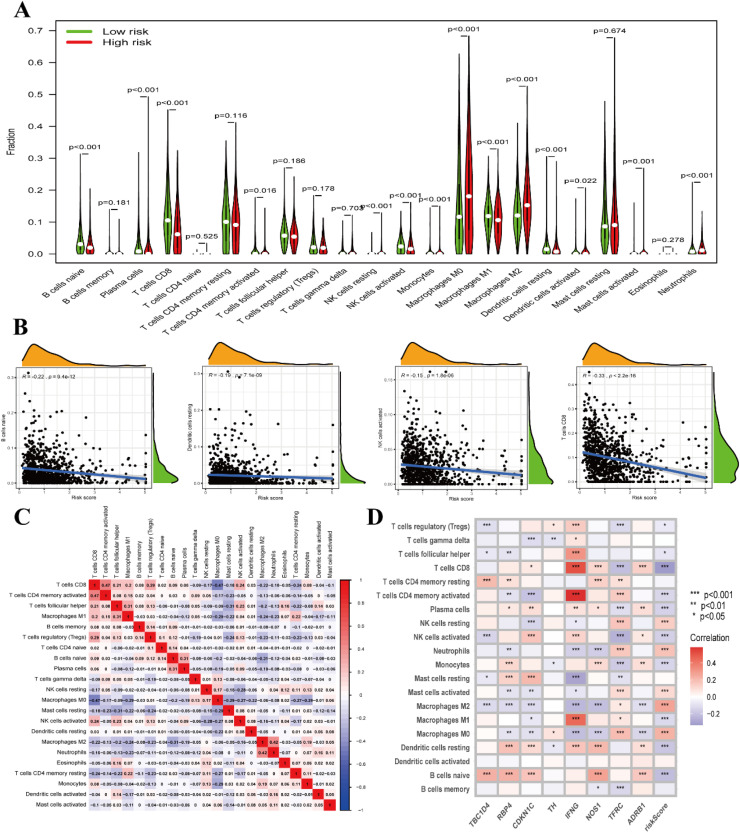
CIBERSORT immune cell infiltration analysis. (A) Differential violin plot of the relative abundance of immune cells in the high and low risk groups. (B) Scatterplot of the abundance of immune infiltration of B-cells naïve, Dendritic cells resting, NK cells activated, and T-cells CD8+.R represents the correlation coefficient and P represents the significance value. (C) Heat map of immune cell correlation. Red represents positive correlations, blue represents negative correlations, and the numbers are correlation coefficients, with higher absolute values of the coefficients resulting in higher correlations. (D) Heatmap of correlations between immune cells and prognostic characteristics associated with diabetes. Red represents positive correlation and blue represents negative correlation; * p < 0.05; ** p < 0.01; *** p < 0.001.

Violin plots (Fig. 7A) and scatter plots (Fig. 7B) depicting immune cell abundance from the CIBERSORT immune cell infiltration analysis revealed significant differences in the relative abundance of B-cells naïve, Plasma cells, T-cells CD8+, T-cells CD4+ memory activated, NK cells resting, NK cells activated, Monocytes, Macrophages M0, Macrophages M1, Macrophages M2, Dendritic cells resting, Dendritic cells activated, Mast cells activated, and Neutrophils between the high and low risk groups. A heatmap (Fig. 7C) displaying immune cell correlations demonstrated that T-cells CD4+ memory activated exhibited the highest positive correlation with T-cells CD8+, while Macrophages M0 displayed the highest negative correlation with T-cells CD8+. The correlation heatmap (Fig. 7D) between our prognostic traits and immune cells revealed that prognostic traits had the highest positive correlation with Macrophages M2 (p < 0.001) and the highest negative correlation with T cells CD8+ (p < 0.001).

### Drug sensitivity analysis

From the results of the drug sensitivity analysis, the authors selected 12 antineoplastic drugs of interest. The graphs (Supplementary Fig. 3A‒D) demonstrate significant differences in drug sensitivity between the high-risk and low-risk groups. Among these drugs, Acetalax, BI-2536, Lapatinib, and OSI-027 exhibited lower half-maximal inhibitory concentration (IC50) values in the high-risk group, indicating that they may be more effective for treating high-risk patients. On the other hand, Cisplatin, Cytarabine, Docetaxel, Erlotinib, Gefitinib, Gemcitabine, Paclitaxel, and Vinorelbine showed higher sensitivity in the low-risk group. This suggests that the former drugs are more suitable for treating high-risk patients, and the latter vice versa. Overall, these findings suggest that our prognostic traits have the potential to predict drug sensitivity.

### Single-cell data analysis

**Fig. 8:**
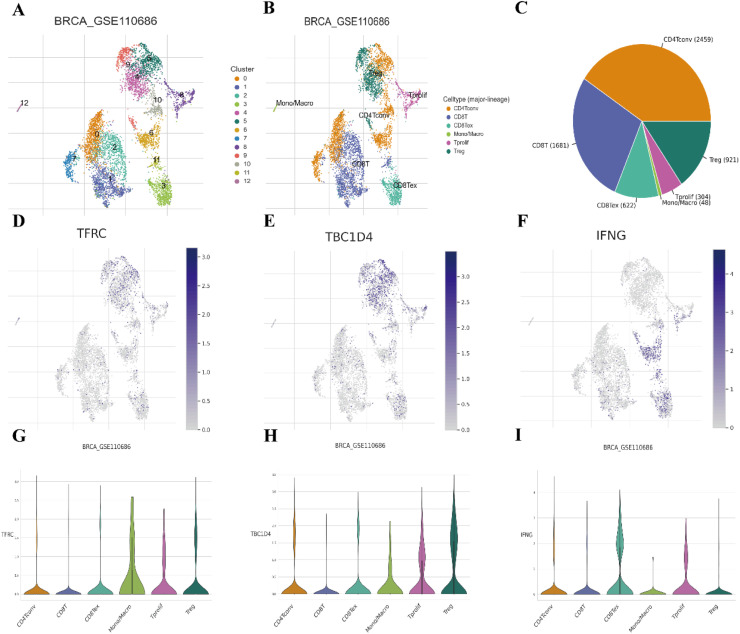
Single-cell data analysis. (A‒B) Unsupervised clustering distribution plots after downscaling of uniform manifold approximation and projection (UMAP) data from GSE110686. (C) Pie charts of Tumor immune single-cell hub 2 (TISCH2) database for different immune cell statistics. (D‒F) Distribution plots of the expression of transferrin receptor (TFRC), TBC1 Domain family member-4 (TBC1D4), and interferon gamma (IFNG) in different clusters, respectively. (G‒I) Expression abundance plots of TFRC, TBC1D4, and IFNG in different expression abundance maps in immune cells.

After performing dimensionality reduction analysis on the UMAP data, the authors classified the BRCA-GSE110686 data from the GEO database into 13 clusters using unsupervised clustering (Fig. 8A). The authors then annotated the BRCA-GSE110686 data into the main six cell types based on specific markers on the cell surface: CD4+ conventional T-cells, CD8+ T-cell, CD8+ Tex cell, Mono/Macro cell, Tprolif cell, and regulatory T-cells (Fig. 8B). The cell counts from the TISCH2 database revealed that the CD4+ conventional T-cells had the highest percentage with 2459 cells, followed by CD8+ T-cell (1681), regulatory T-cells (921), CD8+ Tex cell (622), Tprolif cell (304), and Mono/Macro cell (48) (Fig. 8C).

The authors also examined the expression of the prognostic signature genes TFRC, TBC1D4, and IFNG in the different cell clusters. TFRC was predominantly expressed in CD4+ conventional T-cells and regulatory T-cells, followed by CD8+ T-cells, CD8+ Tex cells, Mono/Macro cells, and Tprolif cells (Fig. 8D‒I). TBC1D4 showed higher expression in regulatory T-cells and CD4+ conventional T-cells, followed by Tprolif cells, CD8+ Tex cells, CD8+ T-cells, and Mono/Macro cells. IFNG exhibited higher expression in CD4+ conventional T-cells, followed by CD8+ Tex cells, regulatory T-cells, CD8+ T-cells, Tprolif cells, and Mono/Macro cells.

Furthermore, KEGG signaling pathway analysis was performed on the BRCA-GSE110686 data. The type I diabetes signaling pathway was found to be significantly up-regulated in CD8+ Tex cells and Mono/Macro cells, and significantly up-regulated in CD4+ conventional T cells and CD8+ T-cells (Supplementary Fig. 4A‒B).

## Discussion

In this study, the authors have successfully established a novel prognostic signature for diabetes-associated 8-RNA based on the TCGA-BRCA and GEO cohorts. The authors have demonstrated the effectiveness and sensitivity of this signature in predicting the prognosis of patients with BRCA using both a test dataset and a validation dataset. Our prognostic trait has also been shown to be an independent prognostic factor, outperforming other clinical traits in terms of predictive performance. To further strengthen the significance of our trait, the authors have created nomogram plots with high accuracy.

Through GSVA analysis, the authors have identified several signaling pathways that exhibit differences. The differential signaling pathways were mainly related to Glycosylphosphatidylinositol (GPI) anchor biosynthesis, JAK-STAT signaling pathway, primary immunodeficiency, T-cell receptor signaling pathway, and PPAR signaling pathway, etc. Several studies have highlighted the close relationship between GPI anchor biosynthesis and diabetes. For instance, T-cadherin, a GPI-anchored cadherin, has been found to be significantly associated with various clinical parameters in diabetic patients.^33^ Additionally, GPI-anchored proteins have shown a positive correlation with blood glucose/insulin levels in rats.^34^ The role of the JAK-STAT signaling pathway in diabetes mellitus cannot be overlooked, as it has been demonstrated to promote the progression of diabetic nephropathy through autophagy in podocytes.^35^ Furthermore, primary immunodeficiency^36^, ^37^, ^38^ and the T-cell receptor signaling pathway^39^ have been directly correlated with the development of type I diabetes. The PPAR signaling pathway also plays a crucial role in diabetic cardiomyopathy, with metformin and glucagon-like peptide-1 agonists as PPAR α-related drugs demonstrating efficacy and safety in lowering lipids and blood glucose levels in diabetic patients.^40^ These studies suggest that the aforementioned signaling pathways are significant in the development of diabetes and may potentially influence the development of BRCA through DRGs. However, further research is required to fully understand the association between DRGs and the development of BRCA.

The authors successfully established a prognostic signature through Lasso regression and Cox regression analyses, consisting of eight DRGs: TBC1D4, RBP4, CDKN1C, TH, IFNG, NOS1, TFRC, and ADRB1. Among these genes, RBP4 is a monomeric binding protein involved in lipid transport and has been found to be dysregulated in various malignancies, including BRCA and lung cancer.^41^ Elevated serum RBP4 levels have been observed in BRCA patients compared to healthy controls.^42^ In mice, RBP4 has been shown to enhance the metastatic potential of BRCA tumors by directly affecting cancer cells and causing endothelial dysfunction and intra-tumor vascular damage^43^ CDKN1C, another gene in our signature, is expressed in the normal epithelium of most BRCA cases, but its expression is reduced at both the mRNA and protein levels in the majority of BRCA cases^44^ CDKN1C is considered a candidate oncogene and plays a crucial role in various human cancers, including BRCA.^45^^,^^46^ Its inhibition in BRCA cells is mainly mediated through histone modification.^47^ Interferon Gamma (IFNG), a pleiotropic cytokine, is involved in the pathogenesis of BRCA^48^ A replication-defective Semliki Forest virus vector SFV/IFNG expressing IFNG has been developed, which induces a therapeutic anti-tumor T-cell response and inhibits tumor growth in a BRCA model.^49^ Additionally, TFRC has been identified as one of the best reference genes for quantifying urokinase fibrinogen activator in BRCA.^50^ Recent studies have shown a 1.586-fold increase in TFRC expression in BRCA tissue compared to normal tissue.^51^ Overall, the DRGs included in our prognostic profile play significant roles in the development of BRCA.

The immune infiltration analysis results revealed significant differences in the relative abundance of immune cells between the high-risk and low-risk groups, with a negative correlation observed with the risk score. This indicates that the risk score accurately reflects the immune status of BRCA patients. Furthermore, our drug sensitivity analysis based on the risk profile successfully identified several anticancer drugs, highlighting the potential of our profile for accurate drug prediction.

In the single-cell sequencing analysis, the authors integrated the expression of prognostic signature genes TFRC, TBC1D4, and IFNG with the clustering results of the six cell types. Interestingly, the distribution of genes characterizing the prognostic features of diabetes consistently pointed towards CD4+ conventional T-cells and regulatory T-cells. Recent studies have shown that in the BRCA signature, Tumor-Associated Macrophages (TAM) can be converted into Tregs by promoting the transition of conventional T-cells to Tregs, which contributes to the accumulation of regulatory T-cells within the tumor.^52^^,^^53^ This finding suggests a potential immune intervention strategy. Additionally, it is worth noting that the type I diabetes signaling pathway was significantly upregulated in CD8+ Tex cells and Mono/Macro cells, while it was significantly downregulated in CD4+ conventional T-cells and CD8+ T-cells. This indicates a strong correlation between the development of diabetes and these specific immune cell types in BRCA patients. These results further validate the strong association between our diabetes-related prognostic profile and the prognosis of BRCA patients at the individual immune cell level. Recent comprehensive studies on advanced-stage breast cancer have highlighted the complex interplay between molecular subtypes, detection methods, and demographic factors in determining patient outcomes.^54^ Our diabetes-related gene signature adds to this evolving landscape by providing a metabolic-immune interface that may help explain the worse prognosis observed in diabetic breast cancer patients. Integrating such signatures with established clinicopathological and molecular classifiers could enhance personalized prognostic models and guide targeted therapeutic strategies.

Our study found that the model's performance varied significantly across different datasets. This variation may be due to data heterogeneity and model overfitting. Specifically, differences between datasets in terms of sample origin, experimental platform/batch, or disease phenotype definition may lead to distribution shifts and potential biases. Additionally, the model may overfit specific dataset noise or patterns during training (especially when model complexity is high or data volume is relatively insufficient), resulting in reduced generalization performance on independent datasets. In summary, enhancing data diversity, conducting rigorous external validation, and adopting strategies to improve model robustness are critical in the application of bioinformatics models.

However, this study has several limitations that need to be addressed. Firstly, the prognostic characteristics identified in this study were not validated through ex vivo experiments, which could have provided further evidence of their accuracy. Secondly, the study utilized retrospective public datasets of varying quality and completeness. Thirdly, the specific mechanism of action of the identified DRGs in BRCA was not experimentally elucidated. Therefore, future studies will focus on investigating the underlying mechanisms to provide valuable insights for researchers in this field.

## Conclusions

This study successfully developed a prognostic signature based on DRGs and systematically evaluated its effectiveness in risk stratification and prognostic prediction for BRCA patients. This signature shows great potential as a valuable biomarker and a possible therapeutic target. Furthermore, our investigation of the expression of the DRGs' prognostic signature in individual immune cells using single-cell sequencing analysis provides a new perspective and a solid foundation for researchers to discover potential targets for immunotherapy.

## Availability of data and materials

This study follows the Transparent Reporting of a Multivariable Prediction Model for Individual Prognosis or Diagnosis (TRIPOD) statement. All datasets used and/or analyzed during the current study are publicly available from TCGA, GEO, and GeneCard database. All data of the independent cohorts in the current study are available from the corresponding authors upon reasonable request.

## Ethics approval and consent to participate

Review and/or approval by an ethics committee was not needed for this study because TCGA and GEO belong to public databases, the patients involved in the database have obtained ethical approval. Users can download relevant data for free for research and publish relevant articles. Our study is based on open-source data and does not involve human or animal experiments, so there are no ethical issues.

## Consent for publication

Not applicable.

## Authors’ contributions

CTZ, XJ, and JYY contributed to the design of the study; XJ, CTZ, YYT, JYL, and JYY contributed to data collection; XJ, JYY, CTZ, YYT, and JYL contributed to data analysis and methodology; CTZ contributed to project supervision; XJ and CTZ contributed to the original draft; and CTZ, XJ, XR, and JYY contributed to revisions of the manuscript.

## Funding

This work was supported by the Shanghai Pudong New Area Science and Technology Development Fund (PKJ2020-Y12).

## Data availability

The datasets generated and/or analyzed during the current study are available from the corresponding author upon reasonable request.

## Declaration of competing interest

The authors declare no conflicts of interest.

## Appendix Supplementary materials

Image, application 1

Image, application 2

Image, application 3

Image, application 4

Image, image 5 dummy alt text

Image, image 6 dummy alt text

Image, image 7 dummy alt text

Image, image 8 dummy alt text
