# Supplementary material for: An 8-gene diabetes-related signature predicts survival and immunotherapy response in breast cancer
Source: Clinics (Sao Paulo). 2026 May 9;81:100986. doi: 10.1016/j.clinsp.2026.100986 (PMC13188118; doi:10.1016/j.clinsp.2026.100986)

**CLINICS-D-25-00756_Supplementary Material**

**Supplementary Table 1** Statistics of clinical information for each data.

|  | **TCGA-BRCA** | **GSE10893-GPL887** | **GSE159956** | **GSE18229-GPL887** |
| --- | --- | --- | --- | --- |
| **Age** |  |  |  |  |
| ≤ 65 | 776 (70.74%) | 45 (48.91%) | NA | 47 (50%) |
| > 65 | 321 (29.26%) | 17 (18.48%) | NA | 17 (18.09%) |
| Unknown | NA | 30 (32.61%) | NA | 30 (31.91%) |
| **Gender** |  |  |  |  |
| Female | 1085 (98.91%) | NA | NA | NA |
| Male | 12 (1.09%) | NA | NA | NA |
| **Stage** |  |  |  |  |
| Stage I | 183 (16.68%) | NA | NA | NA |
| Stage II | 621 (56.61%) | NA | NA | NA |
| Stage III | 249 (22.70%) | NA | NA | NA |
| Stage IV | 20 (1.82%) | NA | NA | NA |
| Unknown | 24 (2.19%) | NA | NA | NA |
| **Grade** |  |  |  |  |
| Grade 1 | NA | 6 (6.52%) | NA | 6 (6.38%) |
| Grade 2 | NA | 15 (16.30%) | NA | 15 (15.96%) |
| Grade 3 | NA | 34 (36.96%) | NA | 36 (38.30%) |
| Unknown | NA | 37 (40.22%) | NA | 37 (39.36%) |
| **T** |  |  |  |  |
| T1 | 281 (25.62%) | NA | NA | NA |
| T2 | 635 (57.89%) | NA | NA | NA |
| T3 | 138 (12.58%) | NA | NA | NA |
| T4 | 40 (3.65%) | NA | NA | NA |
| Unknown | 3 (0.27%) | NA | NA | NA |
| **M** |  |  |  |  |
| M0 | 912 (83.14%) | NA | NA | NA |
| M1 | 22 (2.01%) | NA | NA | NA |
| Unknown | 163 (14.86%) | NA | NA | NA |
| **N** |  |  |  |  |
| N0 | 516 (47.04%) | NA | NA | NA |
| N1 | 364 (33.18%) | NA | NA | NA |
| N2 | 120 (10.94%) | NA | NA | NA |
| N3 | 77 (7.02%) | NA | NA | NA |
| Unknown | 20 (1.82%) | NA | NA | NA |

**Supplementary Table 2** Results of multiple factor Cox regression analyses.

| **Gene** | **Coefficient** |
| --- | --- |
| TBC1D4 | -0.32117 |
| RBP4 | -0.10025 |
| CDKN1C | -0.20901 |
| TH | 0.297607 |
| IFNG | -0.3634 |
| NOS1 | 0.697048 |
| TFRC | 0.206439 |
| ADRB1 | -0.22902 |

**Supplementary Information**

**Additional File 1**

**Figure S1** Least absolute shrinkage and selection operator (Lasso) regression analyses to exclude overfitting genes. (A) Distribution plot of the partial likelihood deviation from the Lasso regression. (B) Cross-validation for tuning parameter selection in the Lasso regression.


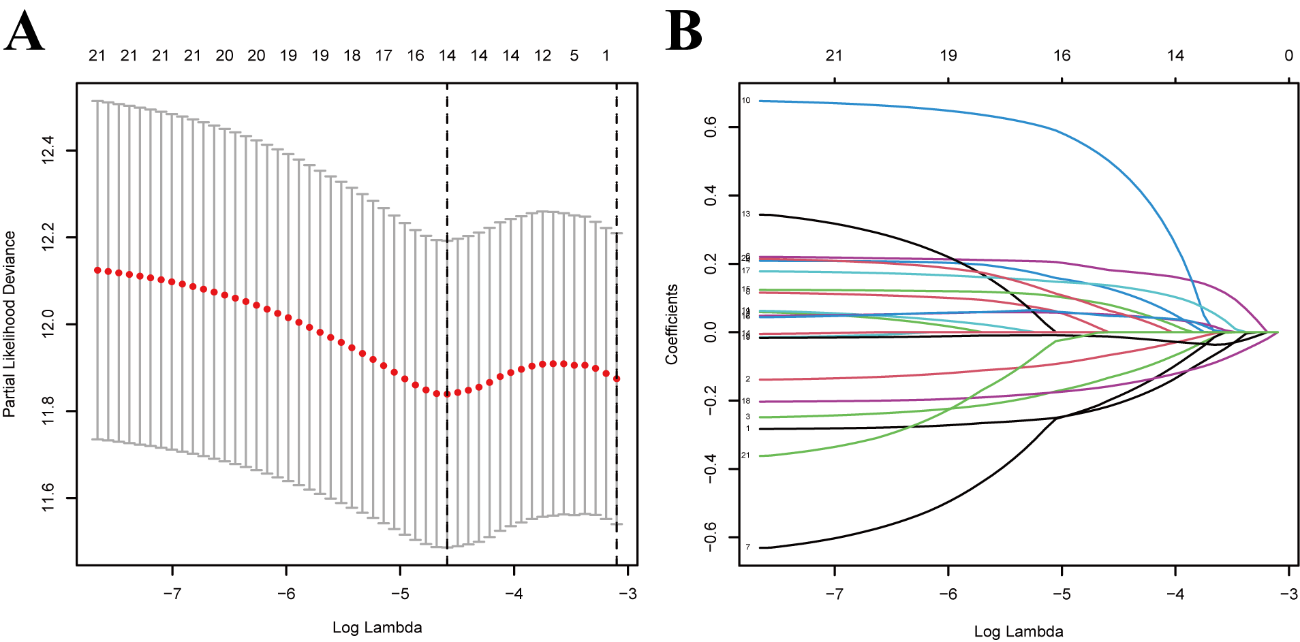


**Figure S2** ROC curve analysis of risk scores and other clinical traits. A Multivariate ROC curve analysis of risk scores and age, gender, stage, T-stage, M-stage, and N-stage.


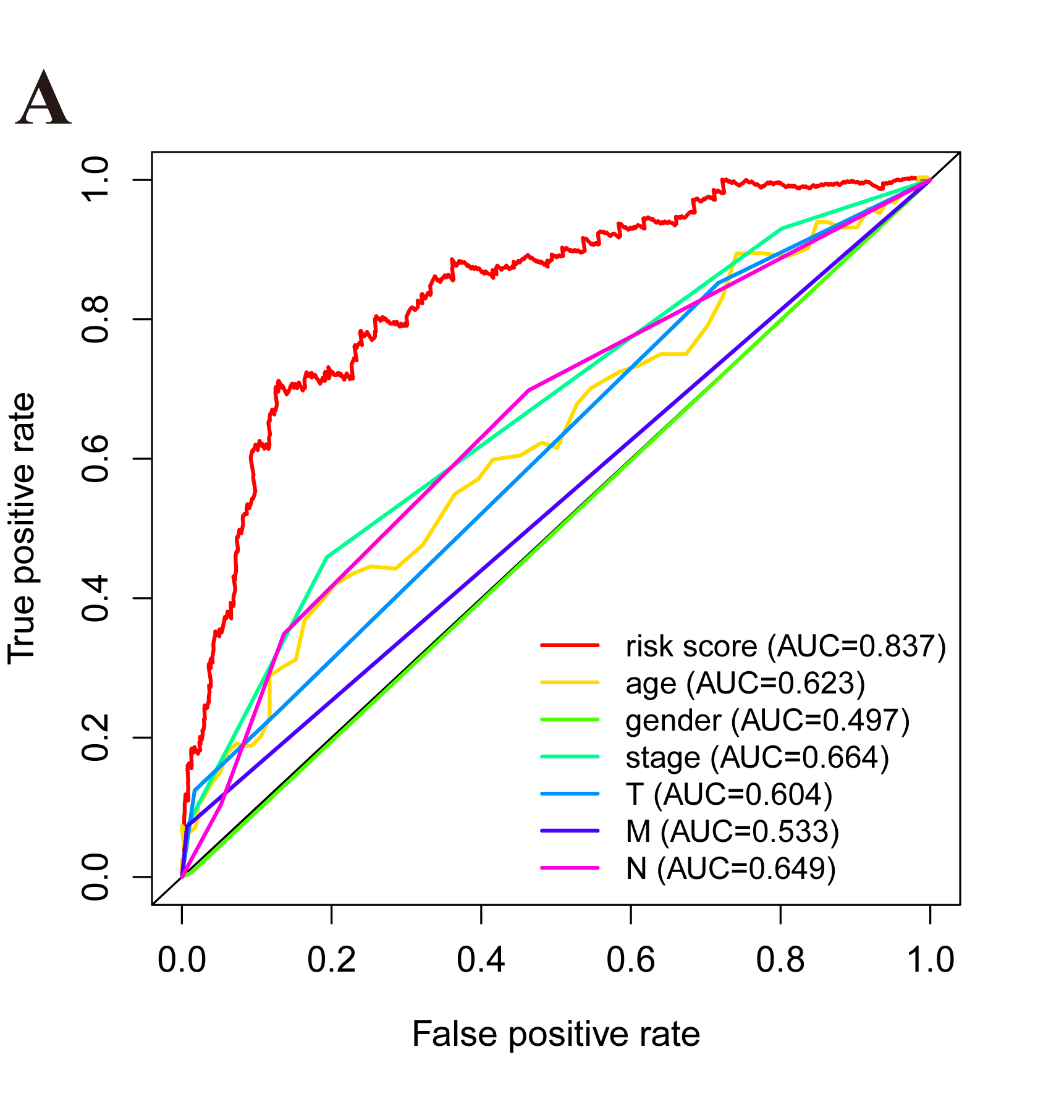


**Figure S3** Drug sensitivity analyses. A-D: Acetalax, BI-2536, Cisplatin, Cytarabine, Docetaxel, Erlotinib, Gefitinib, Gemcitabine, Lapatinib, OSI-027, Paclitaxel and Vinorelbine drug sensitivity box plots in high and low risk groups.


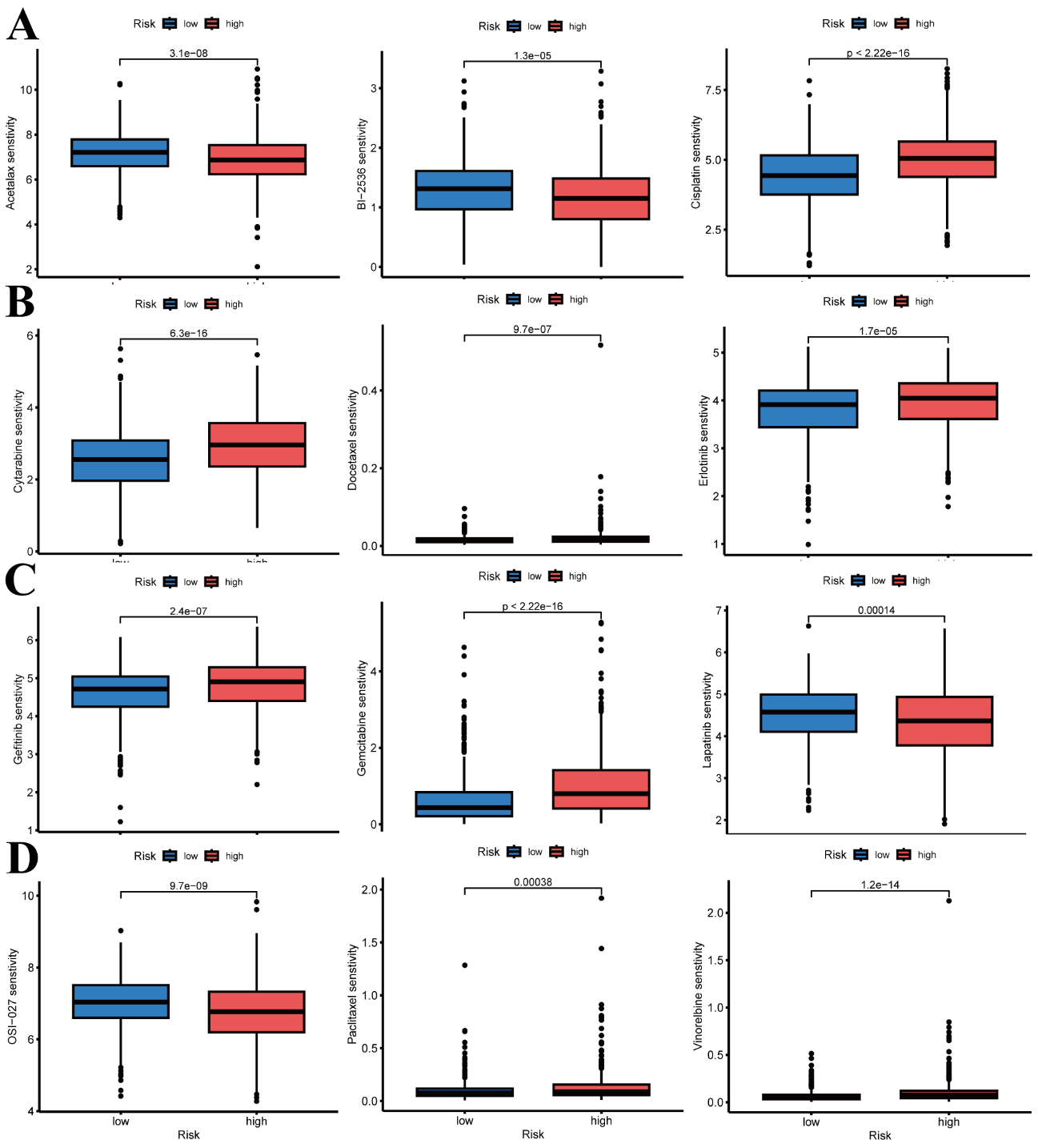


**Figure S4** Kyoto encyclopedia of genes and genomes (KEGG) signaling pathway analysis of BRCA-GSE110686 data. (A) Heatmap of upregulated KEGG gene set. (B) Heatmap of downregulated KEGG gene set.


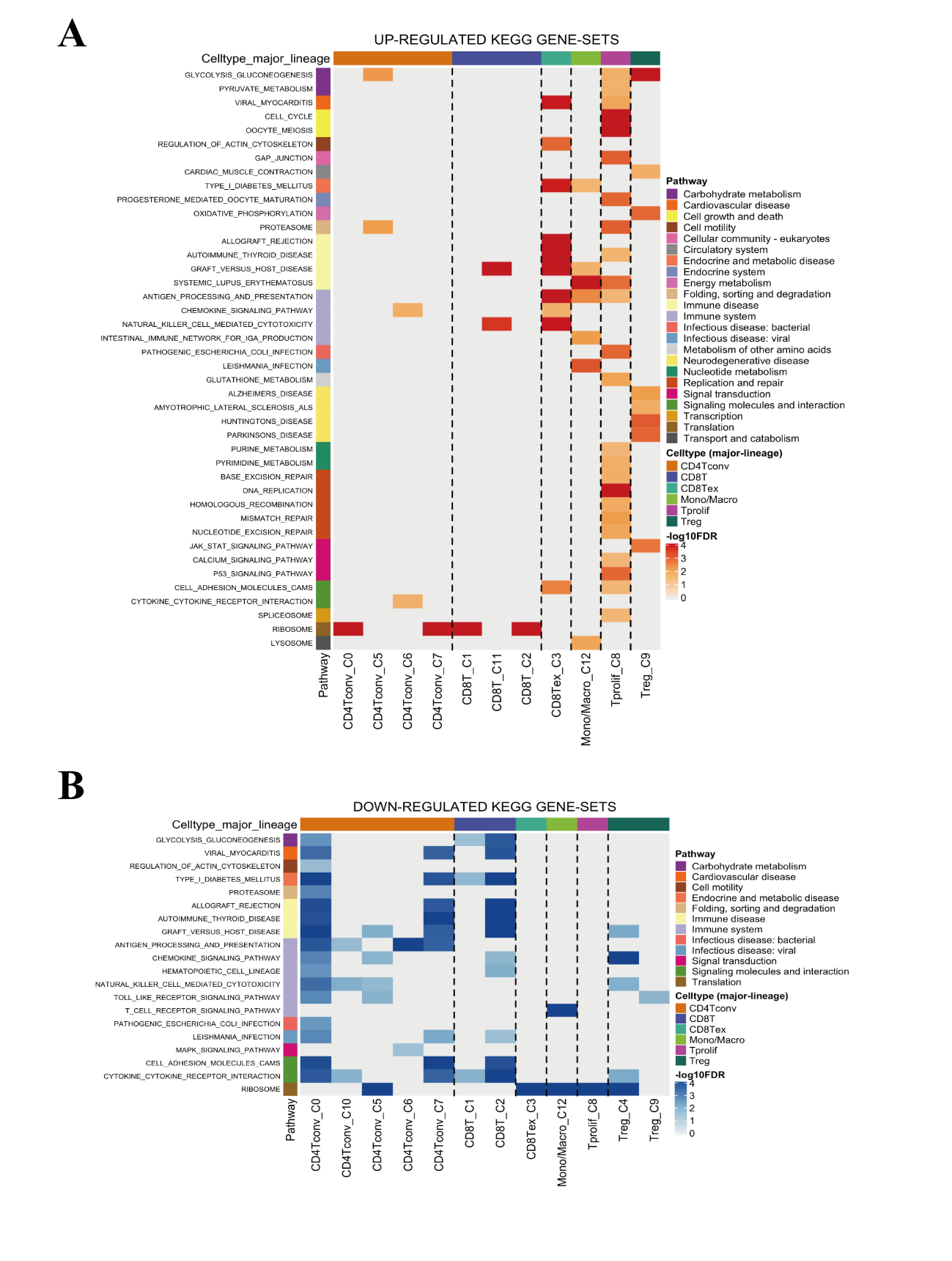

Supplement: Supplementary file 1 [file mmc1.docx]
