# Supplementary material for: An 8-gene diabetes-related signature predicts survival and immunotherapy response in breast cancer
Source: Clinics (Sao Paulo). 2026 May 9;81:100986. doi: 10.1016/j.clinsp.2026.100986 (PMC13188118; doi:10.1016/j.clinsp.2026.100986)
Supplement: Supplementary file 2 [file mmc2.docx]

Supplementary Table 1 Statistics of clinical information for each data.

|  | TCGA-BRCA | GSE10893-GPL887 | GSE159956 | GSE18229-GPL887 |
| --- | --- | --- | --- | --- |
| Age |  |  |  |  |
| <=65 | 776(70.74%) | 45(48.91%) | NA | 47(50%) |
| >65 | 321(29.26%) | 17(18.48%) | NA | 17(18.09%) |
| unknown | NA | 30(32.61%) | NA | 30(31.91%) |
| Gender |  |  |  |  |
| female | 1085(98.91%) | NA | NA | NA |
| male | 12(1.09%) | NA | NA | NA |
| Stage |  |  |  |  |
| stage I | 183(16.68%) | NA | NA | NA |
| stage II | 621(56.61%) | NA | NA | NA |
| stage III | 249(22.70%) | NA | NA | NA |
| stage IV | 20(1.82%) | NA | NA | NA |
| unknown | 24(2.19%) | NA | NA | NA |
| Grade |  |  |  |  |
| grade 1 | NA | 6(6.52%) | NA | 6(6.38%) |
| grade 2 | NA | 15(16.30%) | NA | 15(15.96%) |
| grade 3 | NA | 34(36.96%) | NA | 36(38.30%) |
| unknown | NA | 37(40.22%) | NA | 37(39.36%) |
| T |  |  |  |  |
| T1 | 281(25.62%) | NA | NA | NA |
| T2 | 635(57.89%) | NA | NA | NA |
| T3 | 138(12.58%) | NA | NA | NA |
| T4 | 40(3.65%) | NA | NA | NA |
| unknown | 3(0.27%) | NA | NA | NA |
| M |  |  |  |  |
| M0 | 912(83.14%) | NA | NA | NA |
| M1 | 22(2.01%) | NA | NA | NA |
| unknown | 163(14.86%) | NA | NA | NA |
| N |  |  |  |  |
| N0 | 516(47.04%) | NA | NA | NA |
| N1 | 364(33.18%) | NA | NA | NA |
| N2 | 120(10.94%) | NA | NA | NA |
| N3 | 77(7.02%) | NA | NA | NA |
| unknown | 20(1.82%) | NA | NA | NA |
