# Supplementary material for: An 8-gene diabetes-related signature predicts survival and immunotherapy response in breast cancer
Source: Clinics (Sao Paulo). 2026 May 9;81:100986. doi: 10.1016/j.clinsp.2026.100986 (PMC13188118; doi:10.1016/j.clinsp.2026.100986)
Supplement: Supplementary file 3 [file mmc3.docx]

Supplementary Table 2 Results of multiple factor Cox regression analyses.

| gene | coefficient |
| --- | --- |
| TBC1D4 | -0.32117 |
| RBP4 | -0.10025 |
| CDKN1C | -0.20901 |
| TH | 0.297607 |
| IFNG | -0.3634 |
| NOS1 | 0.697048 |
| TFRC | 0.206439 |
| ADRB1 | -0.22902 |
