# Supplementary material for: An 8-gene diabetes-related signature predicts survival and immunotherapy response in breast cancer
Source: Clinics (Sao Paulo). 2026 May 9;81:100986. doi: 10.1016/j.clinsp.2026.100986 (PMC13188118; doi:10.1016/j.clinsp.2026.100986)
Supplement: Supplementary file 4 [file mmc4.docx]

Xin Jiang

ORCID: 0009-0002-5068-5039

Jianyun Yin

ORCID: 0000-0003-4783-3109

Yingying Tong

ORCID: 0009-0007-6087-8199

Jiayan Li

ORCID: 0009-0005-3114-0617

Xiang Ren

ORCID: 0009-0009-3595-1045

Changtai Zhu

ORCID: 0000-0002-5250-1937
