# Supplementary figures and images for: An 8-gene diabetes-related signature predicts survival and immunotherapy response in breast cancer
Source: Clinics (Sao Paulo). 2026 May 9;81:100986. doi: 10.1016/j.clinsp.2026.100986 (PMC13188118; doi:10.1016/j.clinsp.2026.100986)

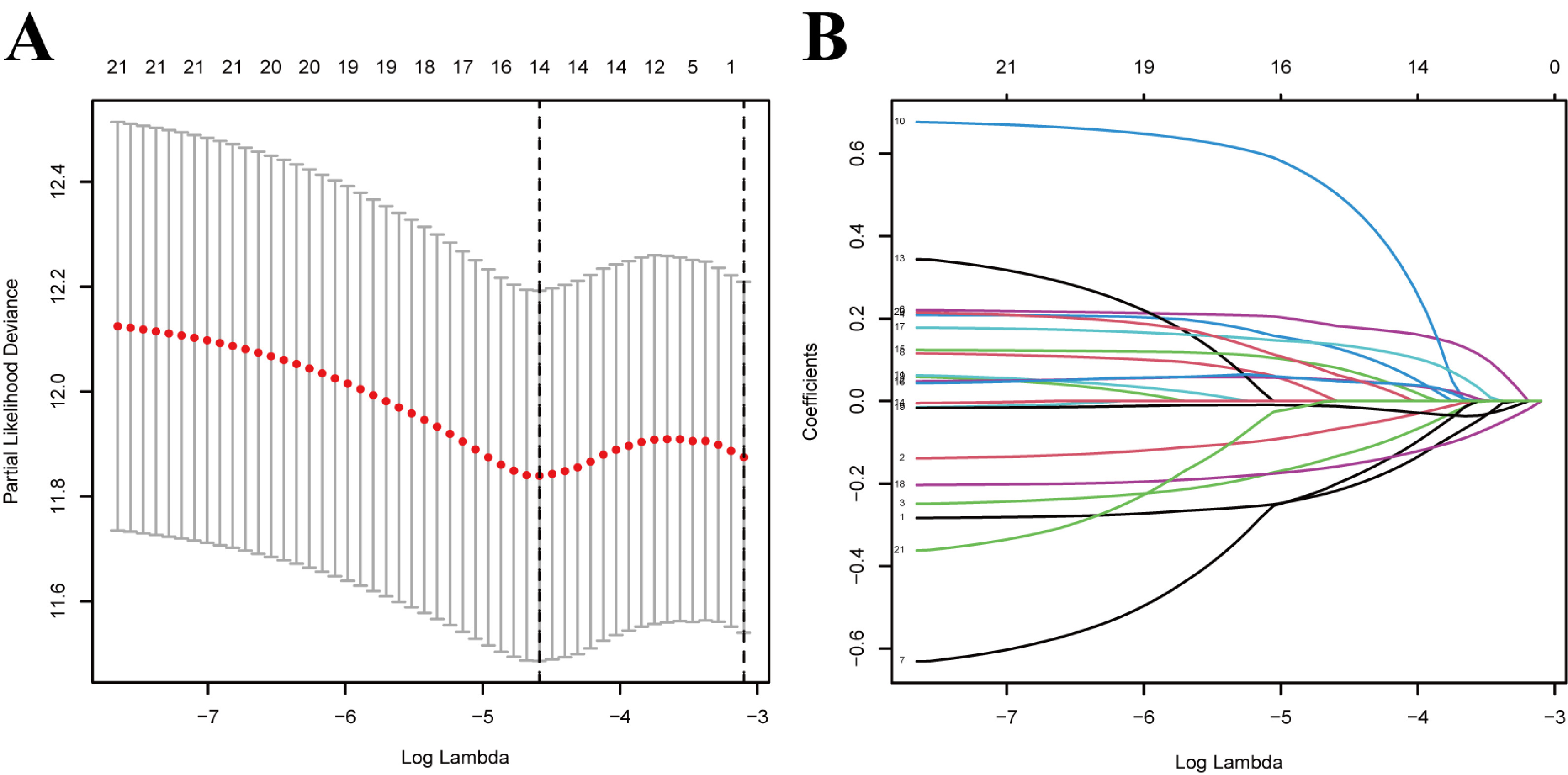

Supplement: Supplementary file 5 [file mmc5.jpg]

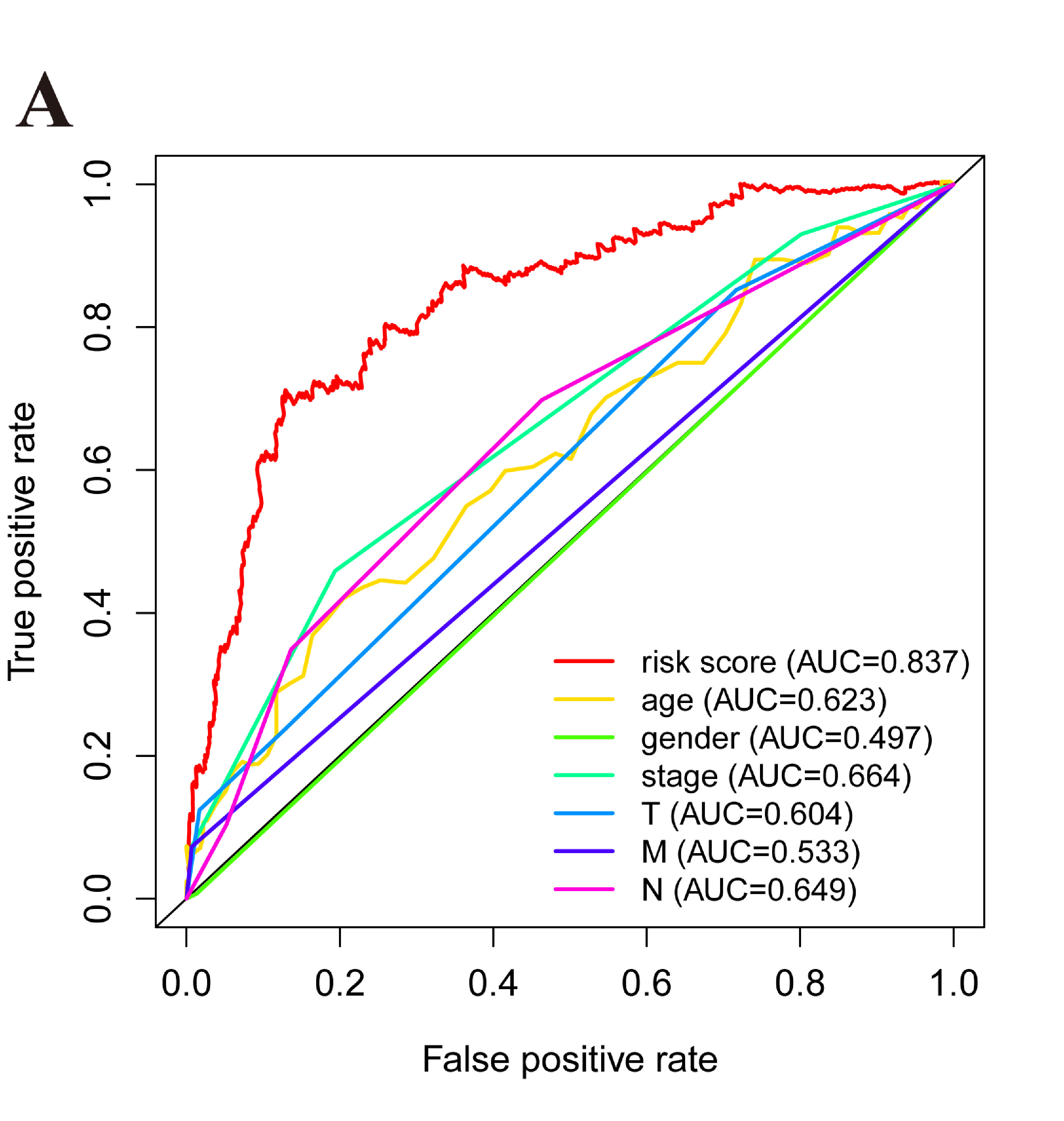

Supplement: Supplementary file 6 [file mmc6.jpg]

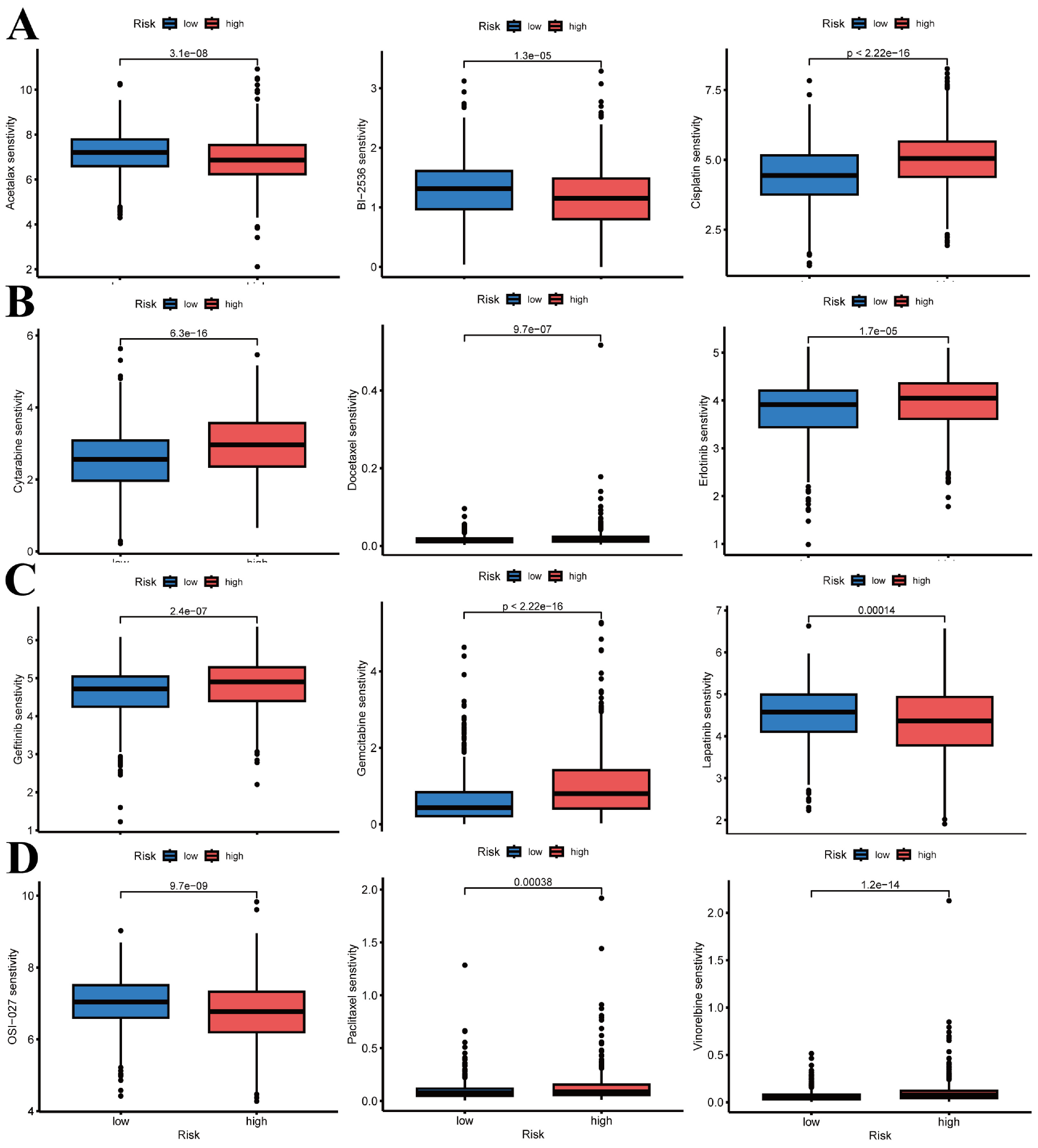

Supplement: Supplementary file 7 [file mmc7.jpg]

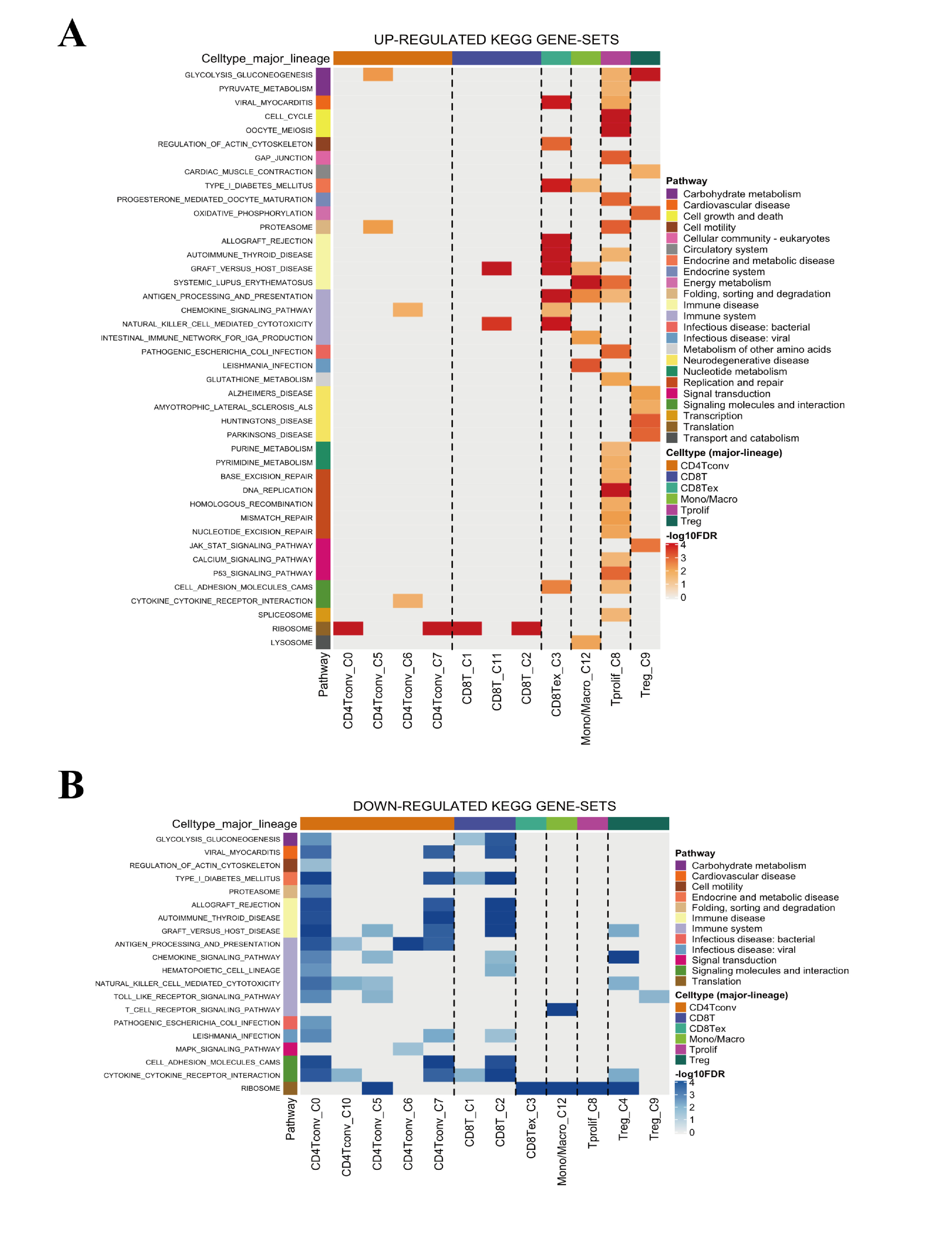

Supplement: Supplementary file 8 [file mmc8.jpg]
